# Direct chemotherapeutic dual drug delivery through intra-articular injection for synergistic enhancement of rheumatoid arthritis treatment

**DOI:** 10.1038/srep14713

**Published:** 2015-10-01

**Authors:** A Reum Son, Da Yeon Kim, Seung Hun Park, Ja Yong Jang, Kyungsook Kim, Byoung Ju Kim, Xiang Yun Yin, Jae Ho Kim, Byoung Hyun Min, Dong Keun Han, Moon Suk Kim

**Affiliations:** 1 Department of Molecular Science and Technology, Ajou University, Suwon 443-759, Korea; 2 Department of Orthopedic Surgery, School of Medicine, Ajou University, Suwon 443-759, Korea; 3 Biomaterials Research Center, Korea Institute of Science and Technology, Seoul 130-650, Korea

## Abstract

The effectiveness of systemic rheumatoid arthritis (RA) treatments is limited by difficulties in achieving therapeutic doses within articular joints. We evaluated the ability of intra-articular administration of injectable formulations to synergistically enhance repair of RA joints. Methotrexate-loaded hyaluronic acid (Met-HA), dexamethasone-loaded microcapsules (Dex-M), and Dex-M dispersed inside Met-HA were prepared as viscous emulsions and injected into articular joints using a needle to form a drug depot. By near-infrared (NIR) fluorescence imaging, we confirmed the local release of NIR from the depot injected into the articular joint over an extended period. In comparison with the subjects treated with Met-HA or Dex-M alone, subjects treated simultaneously with Met-HA and Dex-M exhibited faster and more significant RA repair. Collectively, these results indicated that the drug depot formed after intra-articular injection of Met-HA/Dex-M induced long-lasting drug release and allowed Met and Dex to effectively act in the articular joint, resulting in enhanced RA repair.

Rheumatoid arthritis (RA) is a chronic inflammatory disease with an unknown etiology and a complex multifactorial pathogenesis^1^. RA is characterized by progressive and irreversible inflammatory joint damage that results in the symmetric, progressive destruction of articular joints and bone, and thus severely affects joint structure, joint function, and the quality of life of afflicted patients. Several drugs are available to treat RA. Disease modifying anti-rheumatic drugs (DMARDs) are slow acting drugs that prevent or reduce joint damage^2^. Typical RA treatment involves the use of DMARDs in the incipient stage of the disease.

Methotrexate (Met) is one of the DMARDs most frequently used in the treatment of RA^3^. Systemic toxicity, stomatitis, nausea, bone marrow depression, and liver toxicity limit oral administration of Met due to the repetition Met oral administration ranging from weeks to months^4^. These limitations of Met treatment have prompted research into alternative administration with little side effects.

Dexamethasone (Dex), a synthetic glucocorticoid steroid, has potent immunosuppressive effects and is widely used to treat many inflammatory and autoimmune in addition to RA^5^. However, therapeutic management of long-term pathological conditions with Dex induces unwanted side effects involving the hypothalamic-pituitary-adrenal axis, the cardiovascular system, and fat and bone metabolism^6^. Thus, there is a clear need for a Dex delivery system for the treatment of RA that minimizes associated side effects.

Combination therapies utilizing DMARDs with glucocorticoids have also been shown to reduce the progression of RA articular joint damage^7^. Dex is widely used at the start of RA therapy, in which Dex is administered with Met. Because both Met and Dex can down-regulate expression of several inflammatory genes involved in the pathogenesis of RA, such as tumor necrosis factor-α (TNF-α), interleukin-1β (IL-1β), and IL-6^8^, we hypothesized that RA treatment with a combination of Met and Dex could effectively inhibit the pathogenesis of RA and induce cartilage regeneration *in vivo*.

In recent decades, there has been an increasing focus on intra-articular drug delivery systems^9^,^10^,^11^. A number of attempts using drug-only formulations have been made to maximize local anti-inflammatory effects at the injection site by enhancing exposure of proliferating cells in the articular joint to the drug. However, investigators have found that intra-articular injection of Met or Dex failed to produce significant effects due to the rapid clearance of the drugs from the articular joint^12^,^13^. Thus, to maintain therapeutic concentrations of drugs for prolonged periods, repeated injections into the articular joint are typically required. However, sustained drug concentrations in articular joints for prolonged periods can be achieved by administration of an injectable depot formulation^14^,^15^,^16^,^17^. It is therefore conceivable that injectable Met or Dex depot formulations could improve the local anti‐inflammatory effects of these drugs and induce repair of RA damage.

Hydrogel drug carriers might be suitable for the production of depot formulations of Met for use in articular joints in the incipient stage of RA^18^,^19^,^20^,^21^. Reports suggest that hyaluronic acid (HA), which performs important functions in cartilage, is a good candidate for an injectable depot formulation for intra-articular injection into articular joints^22^. Therefore, we hypothesized that localized injection of a Met-loaded HA hydrogel (Met-HA) could be used to increase exposure of the articular joint to Met, and the *first aim of this work* was to prepare Met-HA for intra‐articular injection into the RA joint to assess RA repair.

Microcapsules have been demonstrated as a suitable carrier for local and long-term drug delivery for Dex^23^,^24^,^25^,^26^, and Dex-loaded microcapsules (Dex-M)^27^, in which Dex is encapsulated in the inner core of the capsule and surrounded by a poly(d,l-lactic-co-glycolic acid) (PLGA) shell, are expected to be a good candidate injectable depot formulation for use in articular joints. Thus, the *second aim of this work* was to show that Dex-M could be utilized for RA repair through intra‐articular injection into affected joints.

In previous studies, our group reported the injectable drug depot of drug-loaded microcapsules dispersed inside hydrogel^28^. Numerous drugs can be easily encapsulated in both the inner cores of the microcapsules and the hydrogels. The drug release from microcapsules could be suppressed and sustained by hydrogel outer shells over an extended period.

When Met and Dex are administered to patients with RA, Met is usually administered in the incipient stage of RA, and Dex treatment is instituted as the disease progresses. In this work we designed the injected drug depot with Dex-M dispersed inside an outer shell of Met-HA. Because Met-HA is a viscous solution at room temperature, Dex-M can be mixed with it easily. HA can control the release of Met and also act as an additional outer shell for Dex-M, and thus extend the release of Dex from the microcapsules. Thus, it was expected that the designed Met-HA/Dex-M formulations could serve as an effective injectable drug depot and thus synergistically enhance local RA repair.

Injectable Met and Dex depot formulation may be the easiest strategy with which to produce sufficient dual drug exposure for proliferating cells in the articular joint over extended periods, and thus to enhance synergistic local RA repair. Based on this concept, the *third aim of this work* was to determine whether intra-articular injection of a combinational dual drug of Met-HA and Dex-M could enhance RA repair through synergistic effects (Fig. 1).

Recently, real time monitoring of *in vivo* drug release can be achieved utilizing noninvasive imaging techniques, including near-infrared (NIR) fluorescence imaging, which can provide real-time imaging data by measuring elimination of autofluorescence from the body^29^,^30^,^31^,^32^. Therefore, we prepared an NIR-loaded injectable formulation to assess the behavior of local release of NIR from a depot injected into the articular joint over an extended period.

## Results

### Preparation of Met-HA and Dex-M

The HA solution has the ideal characteristic of readily incorporating various biological agents by mixing. Thus, Met-HA can be easily prepared as formulation for RA treatment by simply mixing HA with Met. Met-HA was obtained as a translucent, reddish, viscous emulsion that flowed at room temperature when its container was tilted. The viscosity of Met-HA was 1.37 × 10^4^ cP at 37 °C. The IR-780 iodide-loaded HA hydrogel (NIR-HA) for the *in vivo* imaging experiment was also prepared without difficulty using the same method.

A concentric nozzle ultrasonic atomizer was used to form Dex-M as reported previously^25^,^26^,^27^. The atomizer produced spherical microcapsules with a smooth surface structure. The mean particle size of Dex-M was 52 ± 9 μm (Supplementary information). The Dex encapsulation efficiency was 45%. IR-780-loaded microcapsules (NIR-M) were prepared using the same procedure.

Dex-M readily combined with Met-HA to form a mixture of Met-HA and Dex-M (Met-HA/Dex-M) with viscosity of 8.94 × 10^3^ cP at 37 °C. The injectable formulations of Dex-M, Met-HA, and Met-HA/Dex-M were suitable for injection into the articular knee joint using a 21-gauge needle (Supplementary information).

### *In vivo* NIR fluorescence imaging

Noninvasive NIR imaging was used to demonstrate that the tested formulations maintained therapeutic drug levels from the depot injected into the articular joint in treated RA model rats over extended periods.

**Figure 2:**
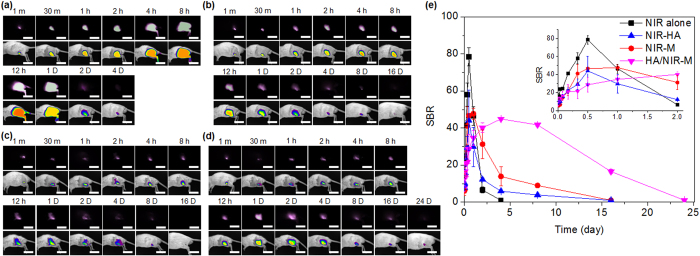
NIR-images after intra-articular injection of (a) IR-780 dye alone, (b) NIR-HA, (c) NIR-M, or (d) HA/NIR-M into articular knee joints of RA animals (Scale bars = 50 mm), and (e) release time versus signal-to-background ratio (SBR) determined at each time point (a–d).

NIR-HA, NIR-M, or a mixture of NIR-M and HA alone (HA/NIR-M) were injected into the articular knee joints of RA rats. A separate group received an intra-articular injection of IR-780 dye alone as a control treatment. All formulations were injected at same NIR amount. No NIR signal was found at the injected group of HA alone, microcapsules alone or mixture of HA and microcapsules. NIR fluorescent images after an intra-articular injection of formulation acquired from the rats are shown in Fig. 2. For all images, high levels of NIR fluorescence were observed at the articular knee joint shortly after injection, and diffusion was apparent after maximum levels in NIR fluorescence were reached. After this point, the intensity and area of fluorescence gradually decreased.

In the rats that received an injection of IR-780 dye alone, the area of NIR fluorescence was increased by diffusion from the injected articular knee joint after the injection, with a maximal area observed 12 h after administration. After 4 days, negligible NIR fluorescence was observed, and no fluorescence was observed after 5 days (Fig. 2a).

Prolonged IR fluorescence was observed in the rats treated with NIR-HA, NIR-M, and HA/NIR-M. NIR-HA and NIR-M showed maximal fluorescence area at 12 h and 24 h, respectively, after treatment, and fluorescence was observed for 8 days, indicating sustained release of NIR. Negligible NIR fluorescence was observed at 16 days (Fig. 2b,c). HA/NIR-M treated-rats showed maximal fluorescence area 4 days after treatment, and fluorescence was observed for more than 24 days, indicating that NIR fluorescence was present for an extended period after HA/NIR-M treatment (Fig. 2d).

For HA/NIR-M, IR-780 dye release was initially inhibited by the microcapsules, causing the lower NIR fluorescence intensity after 12 h in comparison with the rats treated with IR-780 dye alone and NIR-HA. Subsequently, IR-780 dye released from the microcapsules was confined inside the HA hydrogel, resulting in increased NIR fluorescence intensity after 2 days. The controlling effects of the microcapsules and HA on the IR-780 dye extended its release profile for 24 days. Thus, the HA hydrogel acting as an additional outer shell for the microcapsules, as well as the microcapsules, were considered to be important factors influencing IR-780 dye release profiles.

Figure 2e shows the release time versus signal-to-background ratio (SBR) of NIR fluorescence intensity calculated from NIR fluorescent images (see Experimental procedure). At 1 min after intra-articular injection, the SBR of IR-780 dye alone, NIR-HA, NIR-M, and HA/NIR-M showed almost similar values of 6.9 ± 0.2, 9.5 ± 1.1, 7.3 ± 0.2, and 7.3 ± 1.5, respectively. The *T*_max_, *C*_max_, and absolute bioavailability (*AUC*_0–t_) data were calculated from Fig. 2e (summarized in the Supplementary information). The *T*_max_ values of IR-780 dye alone, NIR-HA, NIR-M, and HA/NIR-M were at 12 h, 12 h, 24 h, and 96 h, respectively. In addition, the *C*_max_ values (44–48) of NIR-HA, NIR-M, and HA/NIR-M were significantly lower than that of the IR-780 dye alone (78.5).

The *AUC*_0–t_ values were 0.9 × 10^2^, 1.1 × 10^2^, 2.1 × 10^2^, and 6.3 × 10^2^ for IR-780 dye alone, NIR-HA, NIR-M, and HA/NIR-M, respectively. The relative bioavailability of IR-780 dye from NIR-HA, NIR-M, and HA/NIR-M was 123%, 235%, and 701% of the *AUC*_0–t_ in comparison with the IR-780 dye alone, indicating that the extended release profile led to increased bioavailability of IR-780 dye as a model drug. This result indicated that NIR-HA, NIR-M, and HA/NIR-M were good injectable formulations for the extended release of IR-780 dye.

### Anti-inflammation effects

**Figure 3:**
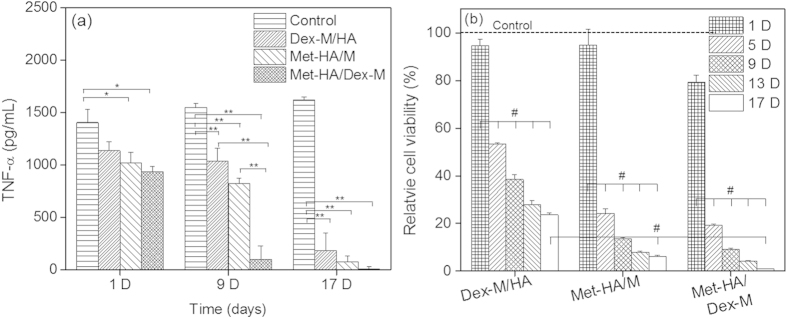
(**a**) TNF-α release amount in RAW 264.7 cells without and with Dex-M, Met-HA, or Met-HA/Dex-M at 1, 9, and 17 days of treatment (*p < 0.05, **p < 0.005). (**b**) Relative cell viability of RAW 264.7 cells at 1, 5, 9, 13, and 17 days of treatment (^#^*p* < 0.001). Dot line represents controls without injectable formulation at each time.

Firstly, to investigate the anti-inflammatory effect of Dex-M, Met-HA, or Met-HA/Dex-M at the cellular level, we measured TNF-α release amount in RAW 264.7 cells (Fig. 3a). The TNF-α release amount of RAW 264.7 cells with no treatment as control was 1,407 pg/mL at 1 day, 1,549 pg/mL at 9 day, and 1,620 pg/mL at 17 day. At 1 day after treatment of Dex-M or Met-HA, the TNF-α release amounts decreased to 1,137 pg/mL and 1,024 pg/mL, respectively. Dex-M and Met-HA maintained similar TNF-α release amounts of 1,039 and 823 pg/mL for 9 days respectively, but rapidly decreased to 186 and 79 pg/mL at 17 days, respectively. Meanwhile, TNF-α release amount of RAW 264.7 cells treated with Met-HA/Dex-M was 940 pg/mL at 1 day and rapidly decreased to 101 pg/mL at 9 day and 9 pg/mL at 17 day, respectively. The extent of TNF-α release in the Met-HA/Dex-M-treated RAW 264.7 cells was significantly lower than those of other formulations.

To examine anti-inflammatory effects, the proliferative activity of RAW 264.7 cells was measured after treatment with Dex-M, Met-HA, or Met-HA/Dex-M (Fig. 3b). A mixture of microcapsules (M) and HA without drugs was used as a control treatment. The relative cell viability exposed to Dex-M/HA in comparison with control decreased from 95% at day 1 to 53% at day 5, and after 9 days gradually decreased to 38–25%, probably due to the extended release of Dex from Dex-M. The relative cell viability exposed to Met-HA/M rapidly decreased from 95.5% at day 1 to 24% at day 5 and to 24–6% as a function of culture time, indicating a significant inhibitory effect on cell proliferation and demonstrating the anti-inflammatory effects of Met-HA over an extended period. Meanwhile, the relative cell viability exposed to Met-HA/Dex-M rapidly decreased from day 1 to day 5. Met-HA/Dex-M showed cell viability to less than 1% at 17 days. Collectively, these results indicated that Met-HA/Dex-M significantly produced anti-inflammatory effects and that increasing incubation time led to greater cell death.

### Confirmation of RA repair via imaging of the feet

**Figure 4:**
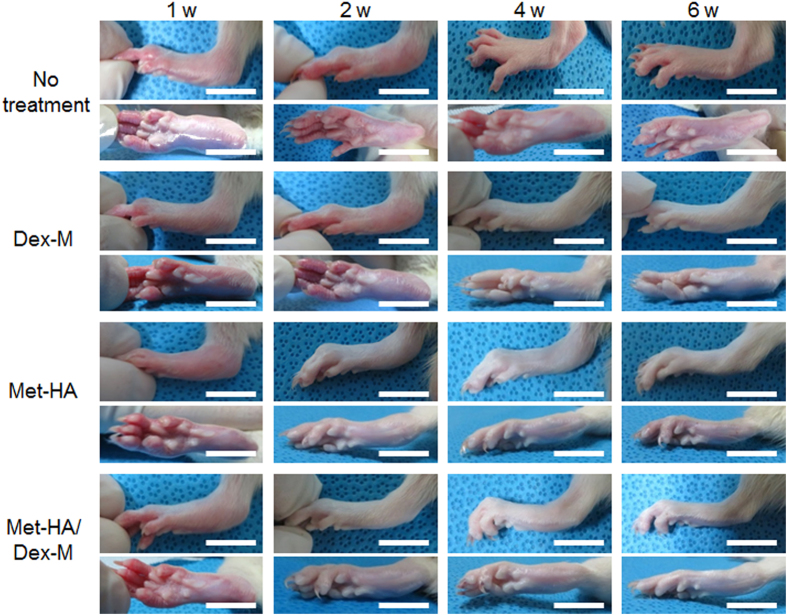
Feet photographs of RA rats after intra-articular injection of Dex-M, Met-HA, or Met-HA/Dex-M at 1–6 weeks (Scale bars = 15 mm). *In accordance with one referee’s suggestion, the experiment for intra-articular injection of free Met was performed at 1, 3, 4 weeks. The feet images showed severe edema and erythema at 1, 3, 4 weeks (Supplementary information), indicating that intra-articular injection of free drug failed to produce significant repair. Thus this result demonstrates that the injectable formulations in this work can improve the therapeutic effectiveness.

Each formulation was prepared for administration to RA animals for *in vivo* assessment of RA repair. Dex-M, Met-HA, and Met-HA/Dex-M were injected into the articular knee joints of RA animals (Fig. 4), which were compared with RA animals that received no treatment by monitoring changes in the tarsals, ankle joints, and feet of the animals.

Untreated RA animals showed severe edema and erythema for 6 weeks. At 2 weeks, RA animals treated with Dex-M showed severe edema and erythema, and RA animals treated with Met-HA showed some edema and erythema. At 6 weeks, RA animals treated with Dex-M or Met-HA showed almost normal feet. Meanwhile, RA animals treated with the Met-HA/Dex-M formulation showed some edema and erythema at 2 weeks and then normal feet at 4 and 6 weeks, indicating that the Met-HA/Dex-M formulation produced more significant repair effects in RA animals than those of Dex-M or Met-HA.

### Confirmation of subchondral bone repair via micro-CT

Bone regeneration in synovial tissue of RA animals treated with Dex-M, Met-HA, or Met-HA/Dex-M was compared with that of the untreated RA animals using micro-CT at five time points: 0, 1, 2, 4, and 6 weeks.

**Figure 5:**
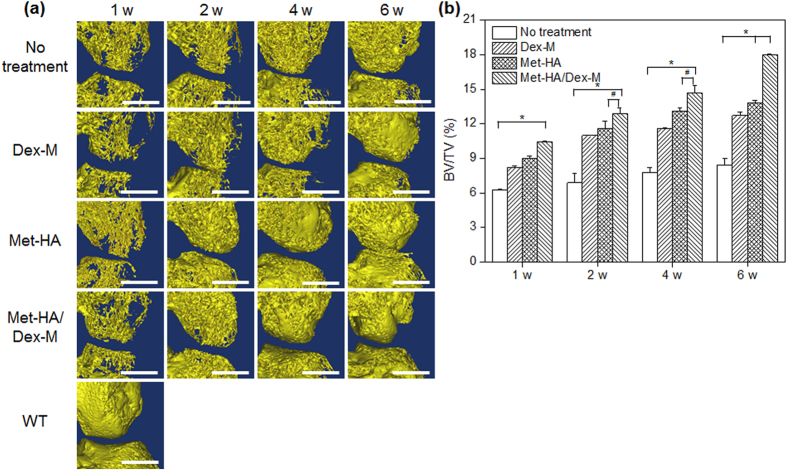
(**a**) Micro-CT images of the articular knee joint of RA animals (Scale bars = 2 mm) and (**b**) volume ratio of neo-bone to tissue (BV/TV) determined from the corresponding CT images after intra-articular injection of Dex-M, Met-HA, and Met-HA/Dex-M at 1–6 weeks (*p < 0.001, ^#^*p* < 0.05).

Figure 5 shows the 3D micro-CT analysis of RA animals, which reveals changes in bone volume for each group. At 1, 2, 4, and 6 weeks post-treatment the defect area was repaired by neo-bone tissue in the articular knee joint in the RA animals treated with Dex-M, Met-HA, or Met-HA/Dex-M. The untreated RA animals showed slight changes in the defect area that were present at the 6-week time point.

The volume ratio of neo-bone to tissue (BV/TV) can be determined from the CT images (Fig. 5b). Neo-bone formation in all treatment groups gradually increased as a function of time. Neo-bone formation in the RA animals treated with Met-HA/Dex-M was more extensive than that of the RA animals treated with Dex-M or Met-HA. There was an increase of 8% in the BV/TV of the untreated RA animals, while the BV/TV of the RA animals treated with Dex-M and Met-HA increased to 13% and 14%, respectively, at 6 weeks (*p < 0.001, ^#^*p* < 0.05). Met-HA/Dex-M showed the highest BV/TV (18%) at 6 weeks.

### Confirmation of RA repair via histology

**Figure 6:**
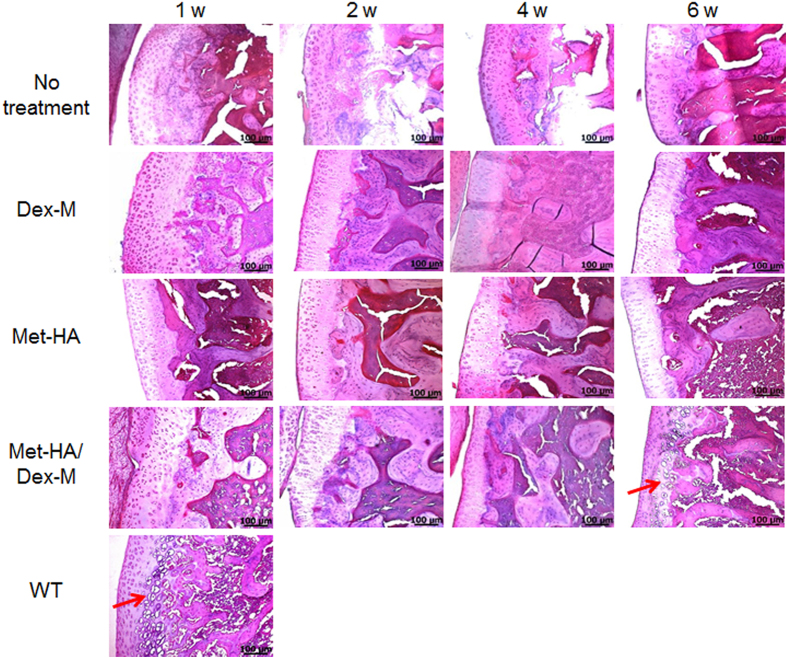
H&E staining of the articular knee joints of RA rats after intra-articular injection of Dex-M, Met-HA, or Met-HA/Dex-M at 1–6 weeks (200× magnification). Arrows show chondrocytes. *In accordance with one referee’s suggestion, the experiment for intra-articular injection of free Met was performed. The images of H&E staining showed almost no chondrocytes at 1, 3, 6 weeks (Supplementary information), indicating that intra-articular injection of free drug failed to produce significant effects owing to the rapid clearance of the drugs from the articular joint. Thus this result demonstrates that the injectable formulations in this work can improve the therapeutic effectiveness.

Synovial tissues in the RA animals treated with Dex-M, Met-HA, and Met-HA/Dex-M were compared with those of the untreated RA animals by assessing images of H&E staining for inflammatory cells, periarticular granulation tissue, and articular cartilage destruction at 1 week (Fig. 6). The untreated RA animals showed almost no change in the H&E staining at any time point. Few chondrocytes were present in the RA animals treated with Dex-M or Met-HA.

In RA animals treated with Met-HA/Dex-M, H&E staining of the tissue at 1 week showed few chondrocytes, but clear staining of chondrocytes within lacunae was observed after 4 and 6 weeks. The chondrocyte population gradually increased as a function of time.

**Figure 7:**
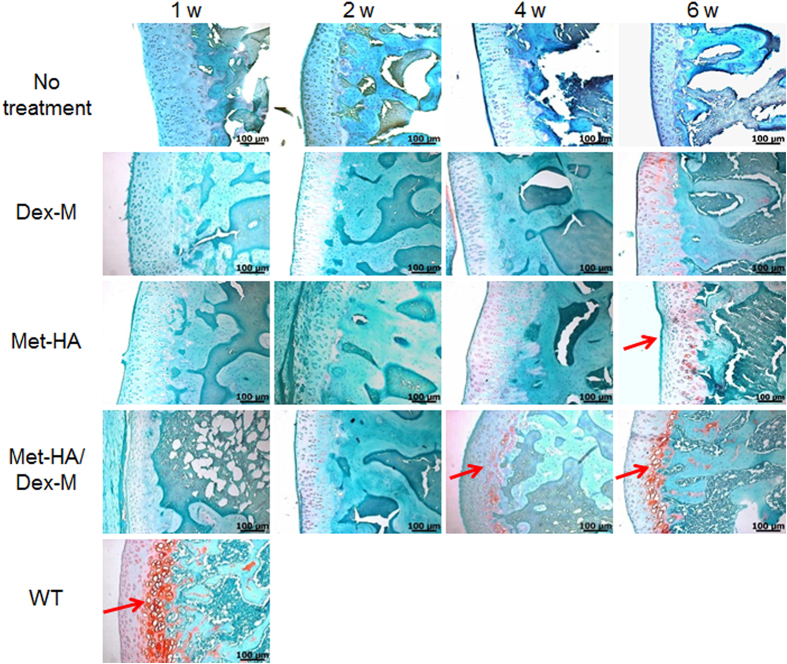
Safranin-O staining of the articular knee joints of RA rats after intra-articular injection of Dex-M, Met-HA, or Met-HA/Dex-M at 1–6 weeks (200× magnification). Arrows show glycosaminoglycan (GAG).

In SO staining (Fig. 7), little positive staining with SO was observed in sections of synovial tissue examined in all treatment groups after 1 and 2 weeks, but subsequently the rounded morphology of mature chondrocytes was surrounded by deposited glycosaminoglycans (GAGs) (deep red color). GAGs were observed at 6 weeks in RA animals treated with Met-HA, indicating the formation of new cartilage-like tissue. For RA animals treated with Met-HA/Dex-M, regeneration of GAGs was observed at the border between the cartilage and the bone at 4 weeks, and the stained area was increased more after 6 weeks. These results show that treatment with the Met-HA/Dex-M dual dosage formulation successfully supports regeneration of cartilage tissue *in vivo*.

**Figure 8:**
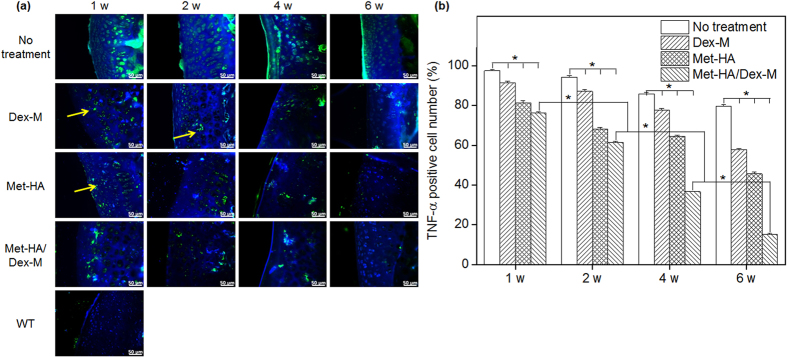
(**a**) TNF-α staining of the articular knee joints of RA rats after intra-articular injection of Dex-M, Met-HA, or Met-HA/Dex-M at 1–6 weeks (400× magnification). DAPI staining (blue) indicates nuclei and arrows indicate TNF-α (inflammation site). (**b**) TNF-α-positive cell counts from the corresponding staining images at 1–6 weeks (*p < 0.001).

Immunohistochemical analysis was performed to examine TNF-α expression in synovial tissue removed from RA animals after treatment with Dex-M, Met-HA, and Met-HA/Dex-M (Fig. 8a). The synovial tissue of normal animals showed no expression of TNF-α, whereas the synovial tissue of RA animals showed abundant TNF-α expression.

For all treatment groups, TNF-α expression was gradually decreased as a function of time. TNF-α expression was observed in RA animals treated with Dex-M or Met-HA after 6 weeks, but at levels that were lower than those observed after 1 week. TNF-α expression was very low in the RA animals treated with Met-HA/Dex-M after 6 weeks.

The proportion of TNF-α-positive cells out of the total cells in the stained tissue area was determined in order to assess the suppression of TNF-α expression in synovial tissue (Fig. 8b). The percentage of TNF-α-positive cells in all treatment groups gradually decreased as a function of time. The percentage of TNF-α-positive cells in RA animals treated with Dex-M or Met-HA decreased to 58% and 46% (*p < 0.001) after 6 weeks, respectively. In RA animals treated with Met-HA/Dex-M, the percentage of TNF-α-positive cells decreased to 76%, 62%, 37%, and 15% (*p < 0.001) after 1, 2, 4, and 6 weeks, respectively. This finding indicated that drug released from Met-HA/Dex-M significantly suppressed TNF-α expression.

## Discussion

RA, a chronic inflammatory disease, is usually treated first with DMARDs, followed by glucocorticoid or nonsteroidal anti-inflammatory drugs^2^,^5^. When RA drugs are administered as systemic treatments, weak therapeutic effects are observed due to the inefficiency of drug transport into the target articular joint^4^,^6^. Some groups have studied intra-articular injection of free RA drugs to increase drug exposure to the articular joint^9^,^10^,^11^. However, RA drugs show rapid clearance from the synovial location, leading to low efficacy due to insufficient exposure of proliferating cells in the joint to the drug. These limitations and drawbacks can be overcome by the administration of a sustained drug delivery system directly to the joint. Recently, several attempts have been made to extend the residence time of RA drugs in the joint cavity.

Therefore, we evaluated whether the injectable formulations prepared in this work can be injected into the articular joint to produce drug depots for extended periods and determined whether the injectable formulations produce synergistic enhanced repair of RA joints after intra‐articular injection. This is the first report of the development of an injectable formulation of Met-HA and Dex-M that is suitable for intra-articular delivery.

In previous studies, our group reported the manufacture of injectable drug depots using drug-loaded microcapsules and injectable hydrogel^18^,^19^,^20^,^21^,^22^,^25^,^26^,^27^,^28^. Numerous drugs can be easily encapsulated in the inner cores of the microcapsules or hydrogels. The formulations of hydrogels and drug-loaded microcapsules can be injected into the body to produce drug depots in a minimally invasive manner, and thus produce a sustained release pattern of the loaded drug over the desired period. Therefore, in this work we chose to study injectable formulations with the aim of producing localized RA drug administration to target articular joints over a prolonged period.

To verify the availability of the RA drugs at the target articular joints after administration of our formulations, we intra-articularly injected NIR-HA, NIR-M, and HA/NIR-M, and conducted NIR imaging in the target articular joint. The evidence for localization of NIR dye in the articular joint was obtained by NIR imaging techniques. NIR fluorescence was maintained in the articular joint for an extended time after a single injection of NIR-HA or NIR-M. NIR-HA showed faster NIR dye release than NIR-M.

In addition, HA/NIR-M, the formulation with NIR-M dispersed inside an HA hydrogel outer shell designed for this work, decreased the initial burst of NIR fluorescence and extended NIR dye release time. The NIR dye release was first controlled by the microcapsules, and subsequently the HA hydrogel drug carrier controlled the amount and duration of released NIR dye at the articular joint. We therefore showed that the injectable formulations designed in this work act as drug depots that produce sustained release of encapsulated products in articular joints.

To avoid systemic toxicity associated with Met and Dex, maintain local therapeutic concentrations in articular joints, and enhance RA repair, intra-articular administration of Met and Dex was adopted in our study. To prepare a pharmacological formulation for the intra-articular injection of Met and Dex, injectable microcapsules and hydrogels were utilized. We prepared injectable formulations of Met-HA and Dex-M as depots for the sustained drug delivery system.

Met was easily mixed into the viscous HA. Dex-M was easily prepared using a concentric nozzle ultrasonic atomizer. Given the NIR imaging result, we also designed a formulation with Dex-M dispersed inside an outer shell of Met-HA to produce Met release in the incipient stage of RA and the subsequent release of Dex, which was prepared by mixing Dex-M and Met-HA.

All formulations were successfully injected into the target articular joint in a minimally invasive manner using a 21-gauge needle. This is the first report of the development of a sustained release dual drug formulation of Met and Dex for intra-articular injection utilizing a drug depot of Dex-M dispersed inside Met-HA.

The RA animals showed no edema and erythema in the feet after treatment with Dex-M, Met-HA, and Met-HA/Dex-M. In particular, the Met-HA/Dex-M dual dosage formulation produced quicker and more significant RA repair. In addition, extensive formation of neo-bone, regeneration of chondrocytes, and GAG deposits were observed after intra-articular injection of Dex-M, Met-HA, and Met-HA/Dex-M. The Met-HA/Dex-M formulation most effectively induced RA repair, followed by the Met-HA formulation, and the Dex-M produced the weakest effect.

TNF-α appears to be critically involved in the initiation and perpetuation of RA^33^. TNF-α is considered to play a prominent role in RA pathogenesis by modulating cell migration, cartilage destruction, and synovial tissue destruction. In this study, severe TNF-α overexpression was observed in the untreated RA animals. RA animals treated with Met-HA and Dex-M showed reduced expression of TNF-α in comparison with untreated RA animals over the course of the experiment. Our results confirm that TNF-α expression in RA is inhibited by Dex or Met released from an intra-articular drug depot. Furthermore, in RA animals treated with Met-HA/Dex-M, mild expression of TNF was observed after 6 weeks due to the long-lasting release of Met and Dex.

Collectively, these results indicated that intra-articular injection of Met-HA/Dex-M allowed Met and Dex to act effectively in the articular joint and induced long-lasting drug release, resulting in enhanced RA repair. Although future studies will be needed to confirm safety of delivery vehicles in other organs, we believe the combination therapy with Met-HA and Dex-M in this work provides a rationale for enhanced RA repair through intra-articular injection.

## Methods

### Materials

PLGA (lactic/glycolic acid = 50/50, MW = 33,000) was purchased from Birmingham Polymers, Inc. (Birmingham, AL, USA). Polyvinyl alcohol (PVA, 87–89% hydrolyzed, MW = 85,000–124,000) was purchased from Sigma-Aldrich (St. Louis, MO, USA) for use as an emulsifier. Bovine type II collagen and complete Freund’s adjuvant used for the animal model were purchased from Chondrex (Redmond, WA, USA). Methotrexate (Met) and dexamethasone (Dex) were purchased from TCI (Tokyo, Japan). IR-780 iodide (NIR dye) was purchased from Sigma-Aldrich (St. Louis, MO, USA). Dulbecco’s modified Eagle’s medium (DMEM), fetal bovine serum (FBS), and penicillin-streptomycin (PS) for culture media were purchased from Gibco (Rockville, MD, USA). Hyaluronic acid (HA) (1.14 MDa) was purchased from Genewel (Seongnam, Korea). All other chemicals were of analytical grade and used without further purification.

### Preparation of the Met-loaded HA hydrogel (Met-HA)

The HA powder was added to 15 mL vials containing phosphate-buffered saline (PBS) and stirred for 4 h to produce 2 wt% HA solution. Methotrexate solution in DMSO was added to the 2 wt% HA solution to produce a final concentration of 375 μg/mL and gently mixed. Met-HA solution was used to treat RA animals. For *in vivo* NIR imaging experiments, IR-780 iodide (375 μg/mL) was added to the HA solution to produce an IR-780-loaded HA hydrogel (NIR-HA).

### Preparation of Dex-loaded microcapsules (Dex-M)

Microcapsules were generated using a mono-axial ultrasonic atomizer (Sono-Tek Corp; Milton, NY, USA). The PLGA and Dex solutions were prepared at concentrations of 3% and 0.3% (w/v) in ethyl acetate, respectively. PLGA and Dex solutions were fed into an ultrasonic atomizer with a mono-axial nozzle at a flow rate of 4 mL/min. Microdroplets were produced by atomizing the mixed solutions of PLGA and Dex within a few seconds at a vibration frequency of 60 kHz and collected in a 2% (w/v) PVA solution for 2 min. The distance between the atomizer head and the aqueous PVA solution was 1 cm, and the stirring speed of the PVA solution was 900 rpm. The resulting solutions were gently stirred at room temperature for 2 h to allow solidification of the microcapsules, followed by filtering and washing with distilled water. The obtained Dex-M was frozen at −83 °C, followed by freeze-drying for 5 days. Just before administration to the RA animals, the Dex-M was dispersed (20%, w/v) in 5% d-mannitol containing 2% carboxymethyl cellulose and 0.1% Tween-80. IR-780 iodide NIR dye was used to produce IR-780-loaded microcapsules (NIR-M) using the same procedure used for the Met-HA formulation.

Scanning electron microscopy was performed with a JSM-6380 SEM (JEOL; Tokyo, Japan) to examine the morphology of the obtained Dex-M. The Dex-M was mounted on a metal stub pre-cooled in liquid nitrogen, after which the metal stub was quickly immersed in a liquid nitrogen bath to minimize alteration of the Dex-M. The stub was freeze-dried at −75 °C using a freeze dryer, and once completely dry the Dex-M on the metal stub was coated with a thin layer of gold using a plasma-sputtering apparatus (Cressington 108 Auto Sputter Coater, Ted Pella Inc.; Redding, CA, USA) under an argon atmosphere.

### Encapsulation efficiency of Dex-M

The encapsulation efficiency of Dex was determined using acetonitrile and 50 mM sodium phosphate. Dex-M (4 mg) was placed into a test tube to which a 1:1 solution of acetonitrile/50 mM sodium phosphate (4 mL) was added to separate Dex from the microcapsules. The resulting mixture was sonicated for 30 min at 25 °C and filtered with a syringe filter. The amount of Dex was determined using a reversed-phase high-performance liquid chromatography (RP-HPLC) system equipped with an Agilent 1200 series HPLC (Agilent; Santa Clara, CA, USA) at 242 nm. The C18 column was a UG120 (250 × 4.6 mm, 5 μm) (Shiseido Fine Chemicals; Tokyo, Japan). The mobile phase was composed of a mixture of acetonitrile and 50 mM sodium phosphate (50:50) and the flow rate was adjusted in the rate 1.0 mL/min. The encapsulation efficiency (*E*) was defined as follows:


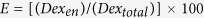


where *Dex*_*en*_ was the amount of encapsulated Dex and *Dex*_*total*_ was the total amount of added Dex.

### Inflammatory tests using RAW 264.7 cells

RAW 264.7 cells were seeded in the bottom of a 24-trans-well plate at a density of 1 × 10^4^ cells/well in culture media consisting of DMEM supplemented with 10% fetal bovine serum and 1% penicillin-streptomycin. After incubation for 24 h, RAW 264.7 cell proliferation was examined in the presence of a mixture of microcapsules (M) and HA without drugs as a control treatment. Dex-M (1.67 mg/mL) with HA, Met-HA (375 μg/mL) with microcapsules, or a mixture of Dex-M (1.67 mg/mL) and Met-HA (375 μg/mL) was added to the wells.

For determining of TNF-α release amounts at 1, 9, and 17 days of exposure, the RAW 264.7 cells were stimulated with 1 mg/mL of lipopolysaccharides (Eccherichia coli 055:B5; Sigma) for 24 h. TNF-α release amounts in the stimulated cell culture supernatant were measured by using mouse TNF-α ELISA Kit (Life technologies, Grand Island, NY) at 1, 9, 17 days.

For the viability of RAW 264.7 cells at 1, 5, 9, 13, and 17 days of exposure, the RAW 264.7 cell viability exposed to each treatment was determined by measuring the conversion of the water-soluble enzyme substrate MTT (3-(4,5-dimethylthiazol-2-yl)-2,5-diphenyltetrazolium bromide) to the purple water-insoluble product formazan in the cytoplasm of viable RAW 264.7 cells. The RAW 264.7 cell viability assessment was performed using a microplate reader (EL808 Ultra Microplate Reader, Bio Tex Instruments; Winooski, VT, USA), and the cell viability of each well was determined at 590 nm. All experiments were performed at least 3 times and the results were presented with mean and standard deviation (SD). The cell viability was individually determined by exposure without and with Dex-M/HA, Met-HA/M and Dex-M/Met-HA at 1, 5, 9, 13, and 17 days. The results were converted into the relative cell viability relating to control at each exposure time which defined as follows;


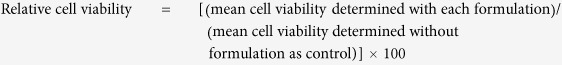


### Preparation of the RA animal model

To prepare the RA animal model, a 1:1 solution of bovine type II collagen and complete Freund’s adjuvant was mixed for 30 min, and 250 μL of the mixed solution (containing 0.5 mg of bovine type II collagen) was injected into the tails of 4-week-old male Lewis rats. After 3 weeks, evidence of edema and erythema was separately observed in the tarsals, ankle joints, knee joint, and feet of the animals.

### Treatment of RA animals

The protocols of this study were approved by the Institutional Animal Experiment Committee (Approval No. 2013-0070) of the School of Medicine of Ajou University. RA treatment experiments were carried out in accordance with the approved guidelines. The three experimental groups received the following treatments: Dex-M (1.67 mg/mL Dex) alone, Met-HA (375 μg/mL Met) alone, or a mixture of Dex-M (1.67 mg/mL Dex) and Met-HA (375 μg/mL Met). Each formulation was individually injected at a volume of 100 μL into the articular knee joint of the RA animals. The RA animals were individually sacrificed at each of the selected post-implantation time points (1, 2, 4, and 6 weeks) to assess the therapeutic effects of the treatments.

### *In vivo* fluorescence imaging

IR-780 iodide dye alone, IR-780-loaded HA hydrogel (NIR-HA), or IR-780-loaded microcapsules (NIR-M) with 375 μg/mL NIR were injected into the articular knee joints of RA animals. At selected times, side-view images of the treated rats were collected at a wavelength of 780 nm using an imaging instrument (FOBI, NeoScience; Suwon, Korea) composed of image sensor of ½” Interline Sony ICX205 1.4 megapixel color CCD with effective pixels of 4.65 μm and frame rate of 15 fps at 1392 × 1040 pixels. For fluorescence excitation, 460 to 730 nm-filtered light was used, and for emission, light was filtered using a 525 to 825 nm band pass filter.

HA hydrogel alone and microcapsules alone showed no fluorescence spectra. In addition, NIR-M also showed no fluorescence spectra owing to its non-solubility in PBS. From fluorescence spectra of NIR alone and NIR-HA in PBS, the extinction coefficient of NIR alone and NIR-HA are 569,400 and 112,100 Lmol^−1^ cm^−1^ at 780 nm, respectively. NIR images are taken with exposure time of 500 sec using dichronic cubes filter (MgF_2_, fused silica filter). At each release time, fluorescence signal intensity and intensity in region of interest in the “signal” or “background” region respectively was determined by using NEOimage software (NeoScience; Suwon, Korea). The signal-to-background ratio (SBR) was calculated at each time point^32^,^34^. All NIR fluorescence images were normalized identically for all conditions of an experiment.

### Microcomputed tomography (micro-CT)

The therapeutic efficiency of each treatment was analyzed by micro-CT carried out with a Skyscan 1076 (Skyscan; Konitch, Belgium) instrument (18.22 μm pixel resolution, 340 ms exposure time, 59 kV energy source, 153 mA current). Approximately 180 projections were acquired over a rotation range of 180°, with a 4° rotation step. The full length of each articular knee joint was scanned, and each joint consisted of 850 slices on average. Three-dimensional virtual models of representative regions in the treated articular knee joints were created and visualized using MIMICS 16.0 software (Materialise’s Interactive Medical Image Control System; Leuven, Belgium).

### Histological analysis

At 1, 2, 4, and 6 weeks after treatment, the rats were sacrificed and each articular knee joint was dissected individually. The removed articular knee joints were immediately fixed with 10% formalin and decalcified using 6% nitric acid (Sigma-Aldrich; St. Louis, MO, USA) for 8 h. The fixed tissues were dehydrated and embedded in paraffin. The embedded specimens were sectioned (5 μm) and the sections were stained with hematoxylin and eosin (H&E), safranin O (SO), and TNF-α stains.

For SO staining, the deparaffinized slides were washed 3 times with deionized water (DW) and treated with Mayer’s hematoxylin solution (Muto Pure Chemicals; Tokyo, Japan) for 5 min, after which the slides were washed with DW for 20 min. The stained tissues were developed with a 0.002% fast green solution (Sigma-Aldrich; St. Louis, MO, USA) for 30 sec and washed with 1% glacial acetic acid solution. The slides were placed in a 0.1% SO solution (Sigma-Aldrich; St. Louis, MO, USA) for 6 min and then fixed with mounting medium (Muto Pure Chemicals; Tokyo, Japan).

For TNF-α staining, the slides were incubated for 10 min at 120–130 °C in citrate buffer solution composed of anhydrous citric acid (Daejung Chemicals; Shiheung, Korea) and anhydrous trisodium citrate (Yakuri Pure Chemicals; Kyoto, Japan), and then washed with PBST (0.05% Tween-80 in PBS). The slides were blocked in PBS containing 5% HS and 5% BSA for 90 min at 37 °C. The slides were incubated at 4 °C for 16 h with the primary antibody (rabbit anti-rat TNF-α, Novus Biologicals; Littleton, CO, USA) in 1% BSA solution (1:200) and washed for 3 h at room temperature with PBS and PBST. Next, the mixture was treated with a secondary antibody (goat anti-rabbit Alexa Fluor® 488, Invitrogen; Carlsbad, CA, USA) (1:1000) and incubated for 3 h. The slides were washed again with PBST, counterstained with DAPI (Sigma-Aldrich; St. Louis, MO, USA) in DW (1:1000), and mounted with a fluorescent mounting solution (Dako; Carpinteria; CA, USA). Immunofluorescence images were obtained using an Axio Imager A1 (Carl Zeiss Microimaging GmbH; Göttingen, Germany) and analyzed with Axiovision software (Rel. 4.8; Carl Zeiss Microimaging GmbH; Göttingen, Germany).

### Data analysis

Cytotoxicity data in RAW 264.7 cells were obtained from independent experiments with *n* = 3 for each data point. Micro-CT data was obtained from independent experiments with the obtained images (*n* = 4). Data are presented as the mean and standard deviation (SD). The results were analyzed using one-way analysis of variance (ANOVA) with Bonferroni’s post-hoc test and SPSS 12.0 software (SPSS Inc.; Chicago, IL, USA).

## Additional Information

**How to cite this article**: Reum Son, A. *et al.* Direct chemotherapeutic dual drug delivery through intra-articular injection for synergistic enhancement of rheumatoid arthritis treatment. *Sci. Rep.* **5**, 14713; doi: 10.1038/srep14713 (2015).

## Supplementary Material

Supplementary Information

## Figures and Tables

**Figure 1:**
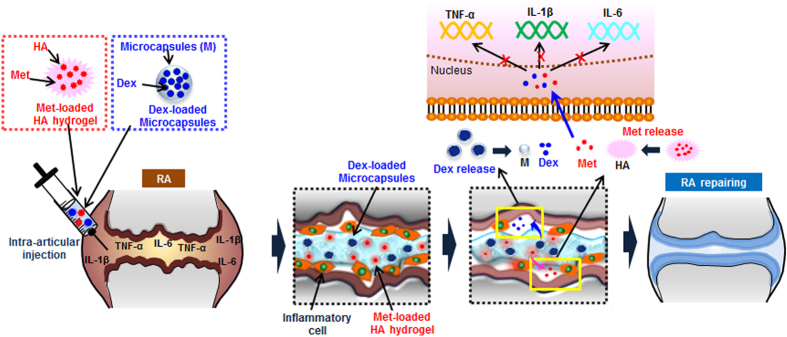
Schematic description of Met-HA/Dex-M injectable formulations for rheumatoid arthritis treatment. (The images were drawn by A.R.S and D.Y.K. using software of Adobe Photoshop7.0).
