# Supplementary material for: Direct chemotherapeutic dual drug delivery through intra-articular injection for synergistic enhancement of rheumatoid arthritis treatment
Source: Sci Rep. 2015 Oct 1;5:14713. doi: 10.1038/srep14713 (PMC4589689; doi:10.1038/srep14713)
Supplement: Supplementary Information [file srep14713-s1.doc]

**Supplementary Information**

**Direct chemotherapeutic dual drug delivery through intra-articular injection for synergistic enhancement of rheumatoid arthritis treatment**

A Reum Son, Da Yeon Kim, Seung Hun Park, Ja Yong Jang, Kyungsook Kim, Byoung Ju Kim, Xiang Yun Yin, Jae Ho Kim, Byoung Hyun Min, Dong Keun Han, Moon Suk Kim*


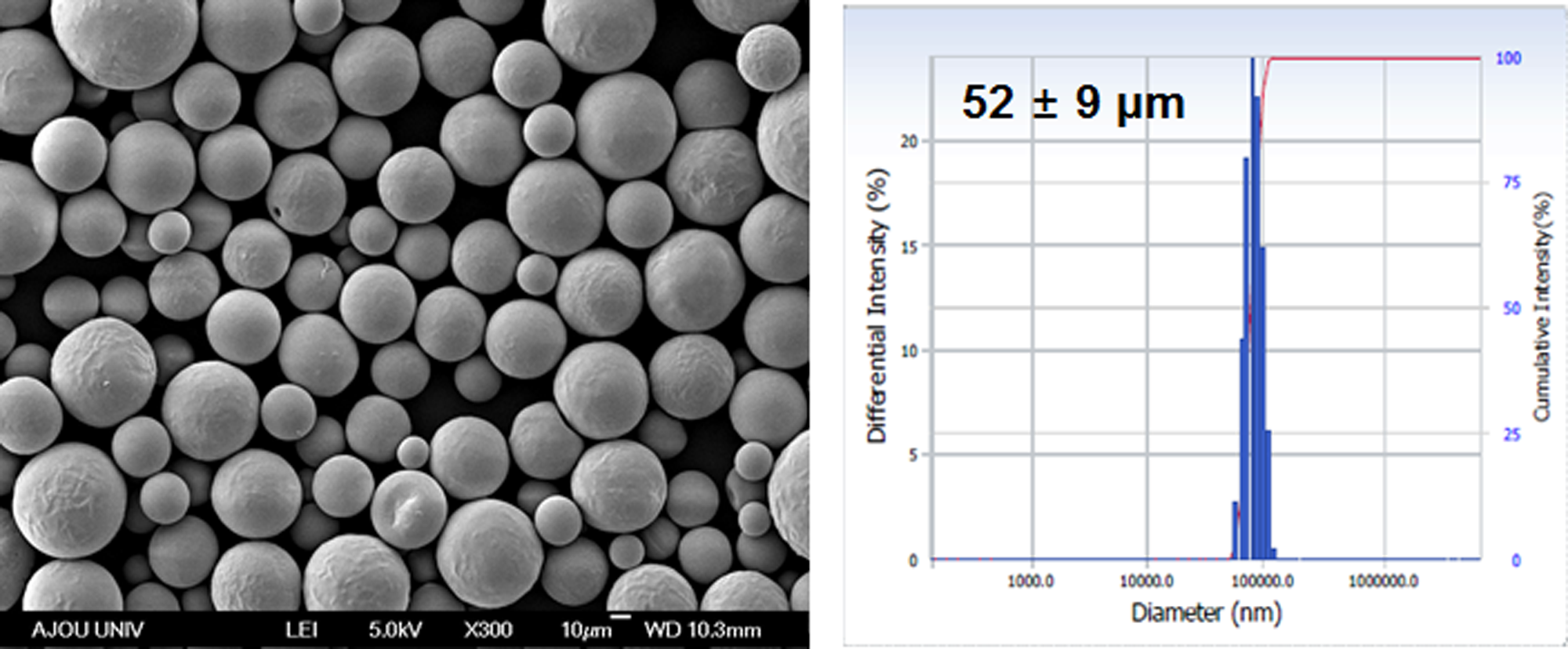


**Figure S1**. (Left) SEM images and (right) particle size measurement of Dex-M.


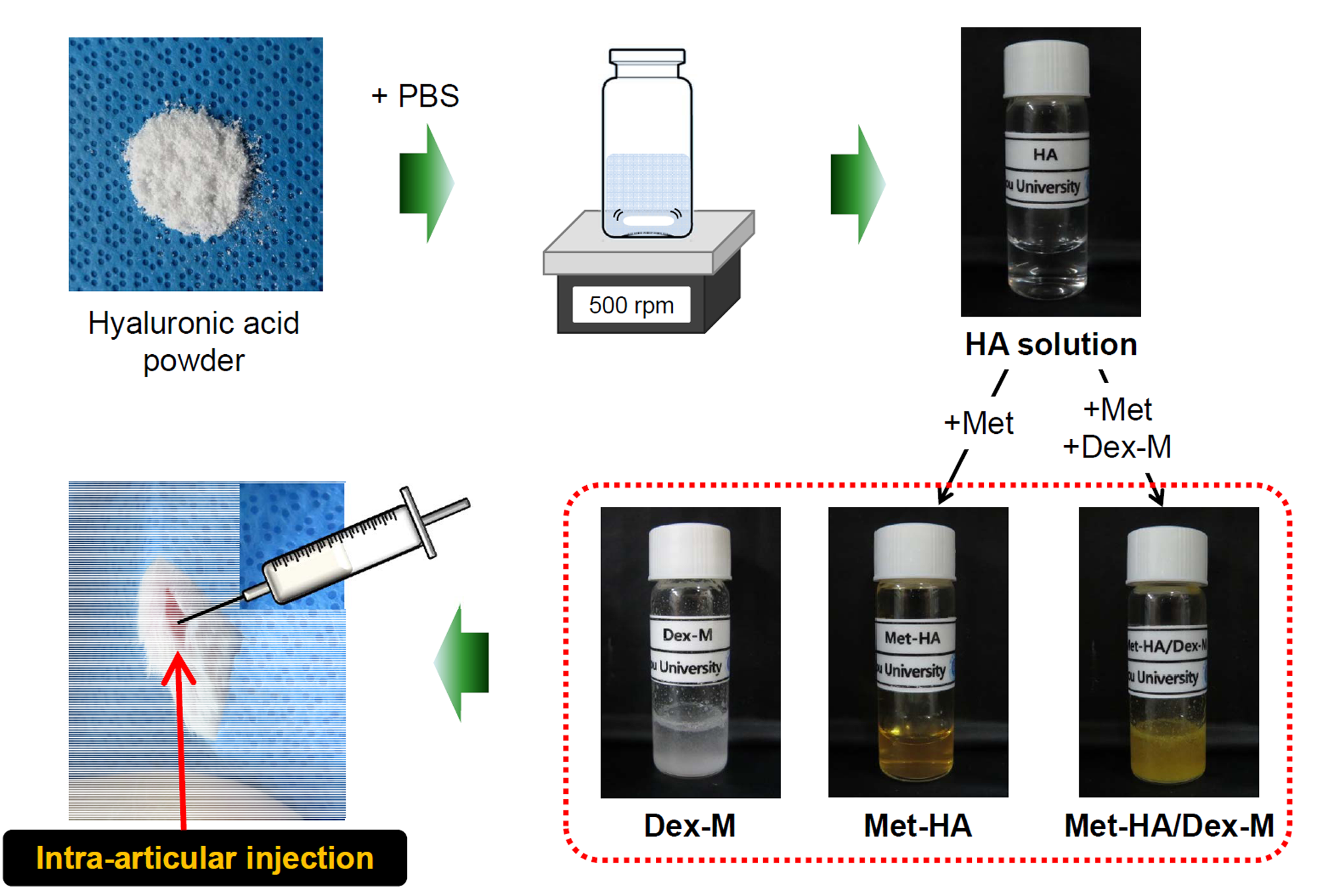


**Figure S2**. Preparation of formulation for RA treatment. (The stirring and syringe images were drawn by D.Y.K. using software of Adobe Photoshop7.0).


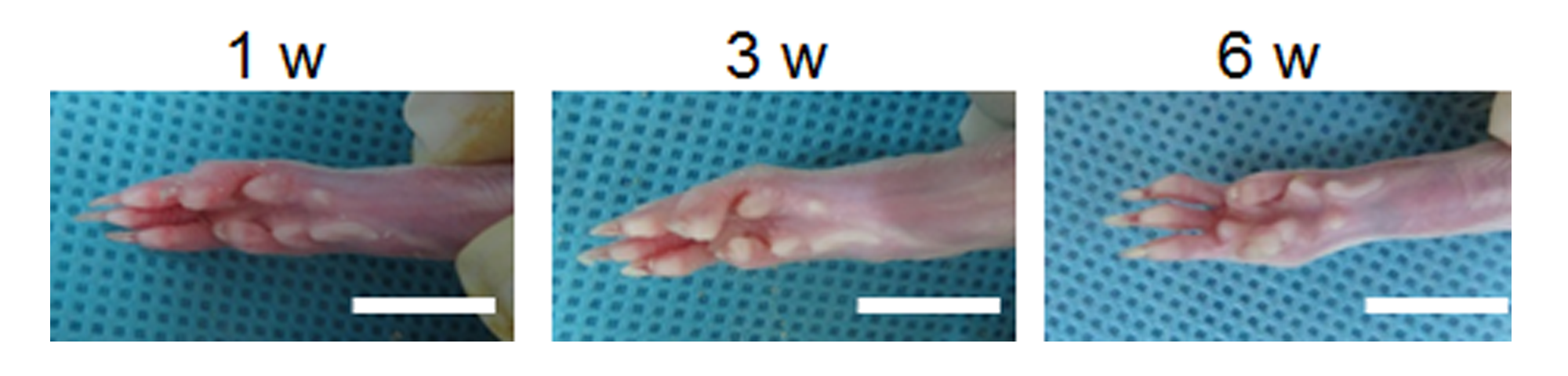


**Figure S3**. Feet photographs at 1, 3, 4 weeks after intra-articular injection of free Met (Scale bars = 15 mm).


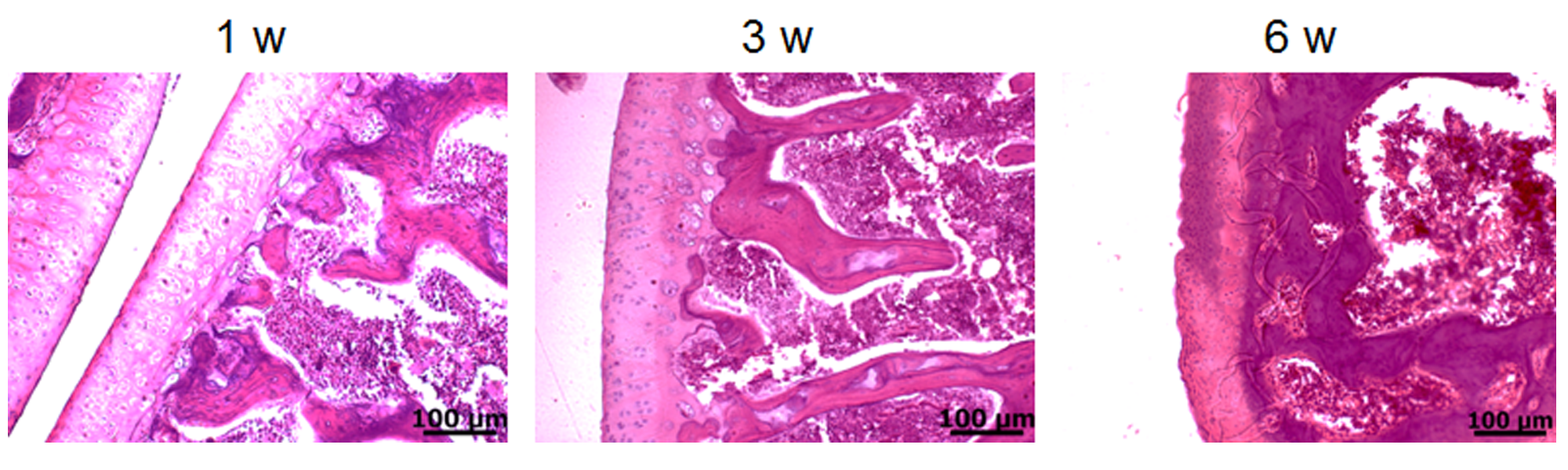


**Figure S4**. H&E staining of the articular knee joints of RA rats after intra-articular injection of free Met at 1, 3, 4 weeks (200x magnification).

**Table S1**. Relative bioavailabilities after intra-articular injection of IR-780 dye alone, NIR-M, NIR-HA, or HA/NIR-M into articular knee joints of RA animals.

| Formulation | *T*_max_ (h) | *C*_max_ | *AUC*_0-t_ | Relative bioavailability (%) |
| --- | --- | --- | --- | --- |
| IR-780  dye alone | 12 | 78.5 | 0.9×10^2^ | 100 |
| NIR-HA | 12 | 44.2 | 1.1×10^2^ | 123.3 ± 13.5 |
| NIR-M | 24 | 47.7 | 2.1×10^2^ | 234.6 ± 0.4 |
| HA/NIR-M | 96 | 44.9 | 6.3×10^2^ | 700.9 ± 21.5 |
